# Melasma: The need for tailored photoprotection to improve clinical outcomes

**DOI:** 10.1111/phpp.12783

**Published:** 2022-03-08

**Authors:** Daniel Morgado‐Carrasco, Jaime Piquero‐Casals, Corinne Granger, Carles Trullàs, Thierry Passeron

**Affiliations:** ^1^ Dermatology Department Hospital Clínic de Barcelona Universitat de Barcelona Barcelona Spain; ^2^ Dermik Clínica Dermatológica Multidisciplinar Barcelona Spain; ^3^ Innovation and Development ISDIN Barcelona Spain; ^4^ Department of Dermatology University Côte d’Azur CHU Nice Nice France; ^5^ University Côte d’Azur, INSERM U1065 C3M Nice France

**Keywords:** dark skin, ethnic skin, melasma, photoprotection, skin of color, sunscreens

## Abstract

**Background/purpose:**

Melasma is a frequent photoexacerbated hyperpigmentary disorder, which can significantly impact on the quality of life. We sought to review the pathogenesis of melasma, and the role of photoprotection in the prevention and treatment of this disorder.

**Methods:**

We conducted a narrative review of the literature. We performed literature searches with PubMed from January 1990 to December 2021 using the keywords “melasma,” “pathogenesis,” “ultraviolet radiation,” “visible light,” “photoprotection,” and “sunscreens.”

**Results:**

The physiopathology of melasma includes a complex interaction between genetics, sex hormones, and sun exposure. Visible light, in particular high‐energy visible light (HEVL), and long‐wave UVA (UVA1) play a key role in melasma pathophysiology, and recent research suggests that melasma shares many features with photoaging disorders. Melasma disproportionately affects dark‐skinned individuals. Some 30% to 50% of South Americans and Asians, among other ethnicities, can present with melasma. Dark‐skinned patients take fewer photoprotective measures. Also, the majority of melasma patients do not adequately follow photoprotection recommendations, including the application of sunscreen. Intensive use of a broad‐spectrum sunscreen can prevent melasma in high‐risk individuals, can lessen melasma severity (associated or not with depigmenting agents), and can reduce relapses.

**Conclusions:**

Due to the physiopathology of melasma, sunscreens should be broad‐spectrum with high sun protection factor, and provide high protection against UVA1 and VL. Sunscreens should be cosmetically acceptable and leave no white residue. Tinted sunscreens are an excellent choice, as pigments can protect from HEVL and UVA1, and may provide camouflage, but they must offer colors that match the skin tone of each patient.

## 1 INTRODUCTION

Melasma is a common photoexacerbated hyperpigmentary disorder characterized by the development of asymptomatic hyperpigmented macules and patches symmetrically distributed on the face. It can be classified according to its distribution pattern into centrofacial (the most frequent), malar, and mandibular.^1^ The physiopathology of melasma includes a complex interaction between genetics, sex hormones (including pregnancy, hormonal therapy, and oral contraceptives) and sun exposure. Visible light (VL), in particular high‐energy VL (400–450 nm) (HEVL), and long‐wave UVA (370–400 nm) (UVA1) play a key role in melasma etiology.^2^ Melasma is observed in all skin types, although it disproportionately affects dark‐skinned individuals, especially women.^3^, ^4^ Some 30% to 50% of Latin Americans and Asians, among other races, can present with melasma.^5^, ^6^ Melasma can significantly impact on the quality of life, even more than vitiligo.^7^ A recent survey performed in Brazil (n=1,518) revealed reduced self‐esteem, and a high incidence of depressive and anxiety disorders among melasma patients, up to 5 times more than in the general population.^8^ Patients can experience a marked improvement in self‐esteem and quality of life after successful treatment of melasma.^9^, ^10^ Patients must be warned that melasma is a chronic disorder that can last for 10 to 20 years, and that treatment of melasma can be challenging and the rate of relapse, extremely high.^11^ Photoprotection is the cornerstone of therapy.^12^, ^13^, ^14^ Unfortunately, multiple studies have shown that dark‐skinned individuals, a high‐risk population for the development of this disorder, take fewer photoprotective measures, including the use of sunscreen.^15^ Due to the physiopathology of melasma, sunscreens for this condition should be broad‐spectrum with high sun protection factor (SPF), and offer high protection against UVA1 and VL.^12^, ^13^ Here, we review the pathogenesis of melasma, and discuss the role of photoprotection as the cornerstone of prevention and treatment of this pigmentary disorder.

## 2 METHODS

We conducted a narrative review of the literature. We performed literature searches with PubMed from January 1990 to December 2021 using the keywords “melasma,” “pathogenesis,” “ultraviolet radiation,” “visible light,” “photoprotection,” and “sunscreens.”

The search was limited to English, Spanish and French language articles. Articles were selected depending on their relevance.

## 3 MELASMA FEATURES EMPHASIZE THE ROLE OF CHRONIC SUN EXPOSURE IN ITS PATHOPHYSIOLOGY

Melasma is a complex disorder associating several pathomechanisms: (1) inappropriate activation of melanocytes, (2) aggregation of melanin and melanosomes in the epidermis and dermis, (3) increased mast cell count and solar elastosis, (4) alteration of the basement membrane, and (5) increased vascularization..^1^, ^2^ Chronic sun exposure plays a critical role in each one of these pathomechanisms.^1^, ^2^ Interestingly, in a very large epidemiologic study, the risk of onset during pregnancy was associated with having spent more time outdoors (an extra 10 h per week spent working outside increases the odds of onset of melasma during pregnancy by approximately 27%).^4^ The same study reported a significantly earlier onset of melasma in fair skinned patients compared to dark skin types.^4^ Melasma affects not only melanocytes but also keratinocytes, fibroblasts, mast cells, endothelial cells and possibly sebocytes.^2^, ^3^ Increased dermal mast cells and solar elastosis, together with an altered basement membrane and increased vascularization are the hallmarks of photoaging, and melasma could be considered as a photoaging skin disorder occurring on a predisposed genetic background, rather than simply a pigmentation disease.^2^ Furthermore, Raman spectroscopy measurements have revealed protein breakdown and molecular degradation in melasma lesional skin, another sign of photodamage.^16^ Further, fibroblasts isolated from photoaging skin or *in vitro* photoinduced senescence‐like phenotype of fibroblasts produce an increased number of promelanogenic growth factors.^2^, ^17^

**FIGURE 1:**
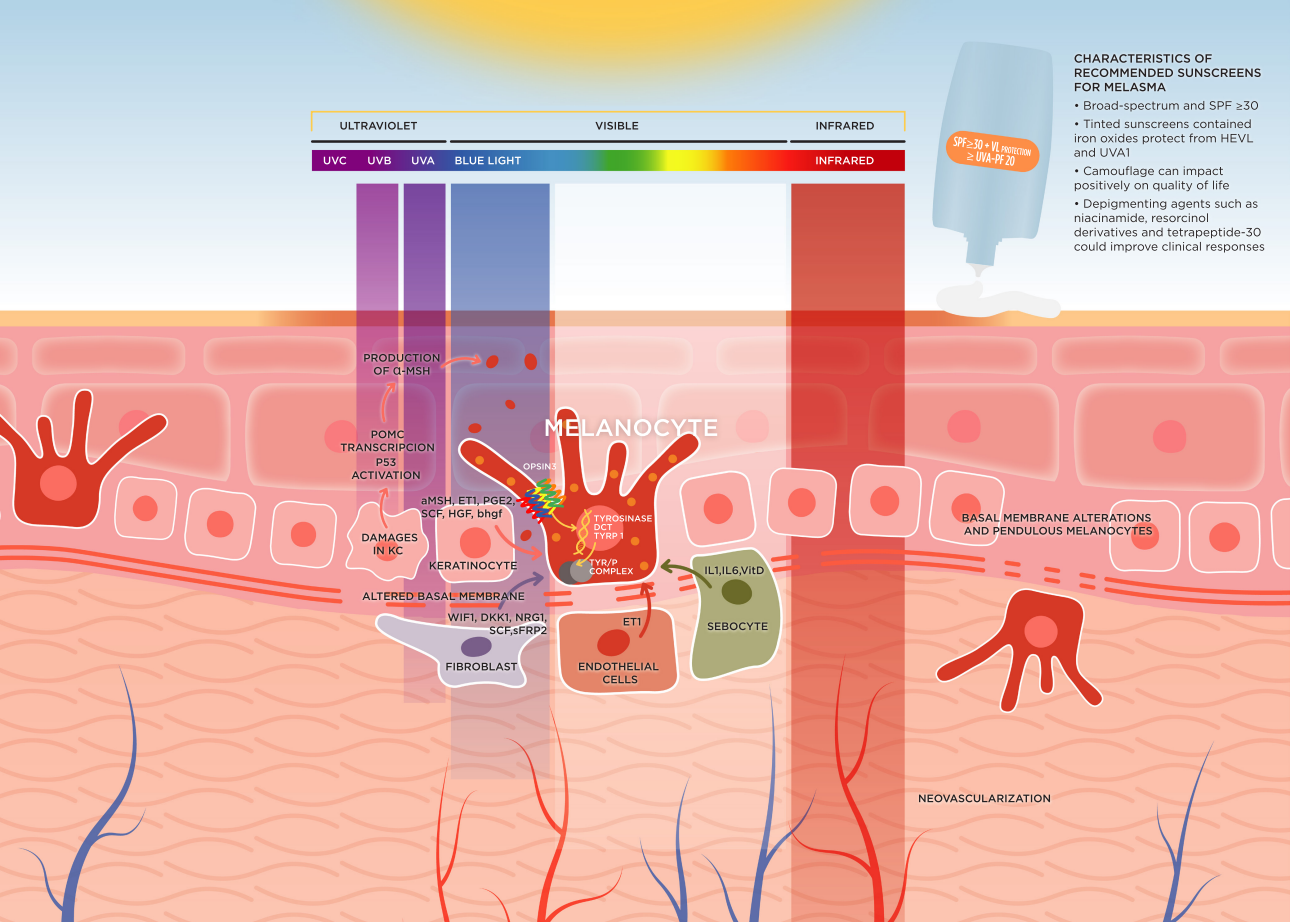
Diagram summarizing pathologic changes and mechanisms in melasma. Melanocytes are producing and distributing melanin to surrounding keratinocytes. UVB‐induced DNA damage of keratinocytes activates P53, binding to the POMC promoter and finally leads to the secretion of α‐MSH by keratinocytes. Chronic exposure to UVA and VL alters the dermal component. VL induces skin pigmentation through the activation of Opsin 3, a photoreceptor, which mediates the expression and activity of the rate‐limiting enzyme tyrosinase in melanocytes. Basal membrane alteration promotes the descent of melanocytes to the dermis (pendulous melanocytes). Keratinocytes produce cytokines and hormones, especially after UVB exposure. Fibroblasts secrete factors that influence melanogenesis and melanocyte proliferation. The increased vascularization plays a key role, especially with endothelial cells that produce ET1, which is a potent activator of melanogenesis. Abbreviations: DCT dopachrome tautomerase; DKK1, Dickkopf 1; DNA, Deoxyribonucleic Acid; ET1, Endothelin 1; HGF, hepatocyte growth factor; KC, Keratinocytes; p53, Tumor protein p53; PGE2, prostaglandin‐E2; POMC, Proopiomelanocortin; SCF, stem cell factor; sFRP2, Frizzled‐related protein 2; SPF, Sun Protection Factor; TYR/P COMPLEX, tyrosinase‐related protein complex; TYRP1, tyrosinase‐related protein 1; UVA, Ultraviolet A; UVB, Ultraviolet B; UVC, Ultraviolet C; WIF1, Wnt inhibitory factor 1; α‐MSH, α‐Melanocyte‐stimulating hormone

The effects of radiations of longer wavelengths and deeper penetration in the dermis than UVB, including UVA1 and VL (400–700 nm), have been highlighted in the pathogenesis of melasma in recent years^2^, ^13^ (Table 1) (Figure 1). HEVL alone or in combination with infrared radiation can generate reactive oxygen species (ROS), increase matrix metalloproteinases expression, collagen degradation, and induce indirect DNA damage^18^, ^19^, ^20^; oxidative stress also contributes to pigmentation.^21^ Furthermore, HEVL can induce both immediate and persistent hyperpigmentation in higher skin phototypes (III‐IV),^22^, ^23^, ^24^ but not in phototypes I and II. Effective melanogenesis after irradiation with HEVL has been observed in the basal layer of affected and non‐affected skin of patients with melasma (skin phototypes III‐V).^25^ The combination of HEVL and UVA1 acts synergically, and can produce hyperpigmentation and inflammation in skin phototypes IV‐VI,^26^ and erythema in skin phototypes I‐II.^27^ The activation of the photoreceptor Opsin 3 by HEVL, which mediates the expression and activity of the rate‐limiting enzyme tyrosinase in melanocytes, is the proposed molecular mechanism of the pigmentation induced by this radiation.^28^

**TABLE 1:** Detrimental effects of visible light

Inflammatory responses	Generation of reactive oxygen and nitrogen species Increased proinflammatory cytokines and activation of matrix metalloproteinases Indirect DNA damage Damage to the skin barrier function
Melanogenesis	Induction of immediate and prolonged pigmentation in dark‐skinned individuals* Exacerbation of preexisting hyperpigmentation/melasma
Vascular	Dilatation of vessels of the subpapillary plexus, and induction of erythema
Aggravation of photoinduced disorders	Solar urticaria, polymorphous light eruption, cutaneous porphyrias and chronic actinic dermatitis

*Fitzpatrick skin phototypes III to VI.

## 4 PHOTOPROTECTION PRACTICES IN MELASMA PATIENTS AND POPULATIONS AT RISK

Melasma has a high prevalence among dark‐skinned individuals, both women and men.^29^ In a large Indian study (*n* = 1,204), 30% of women presented with facial melasma.^5^ In Mexico, melasma affected up to 50% of pregnant women and accounted for 4%–10% of new dermatology hospital referrals.^6^ Multiple studies have shown that dark‐skinned individuals take fewer photoprotective measures, including the use of sunscreens.^15^, ^30^, ^31^ Even among individuals at high‐risk for melasma such as pregnant women in Brazil, the daily use of sunscreen can be low (<30%). Similar results have been reported in studies among dark‐skinned individuals with melasma or other disorders of hyperpigmentation: the rate of sunscreen use is only 35%‐ 67.5%,^3^, ^32^ and its intensive use (reapplication every 2 h) is even lower: 7.6%.^32^ In a large epidemiologic study on 324 women from nine countries in three continents, only one third of melasma patients reported changing their behavior regarding sun exposure and protection after the onset of melasma, including only a slight increase in the use of sunscreen after onset.^4^ Lack of education on photoprotection for dark‐skinned individuals, misconceptions and popular myths (“dark skin does not get sunburnt”) can be frequent among patients and clinicians.^33^ In a recent survey, more than half of the dermatologists stated that they counsel dark‐skinned individuals less on sunscreen use than light‐skinned individuals.^30^ In a study on patients with dyschromia, dermatologists prescribed sunscreens for dark‐skinned individuals less frequently. Sunscreens were the third most common treatment for dyschromia in Caucasians, the sixth in African Americans, and the tenth in Asians.^34^

## 5 PHOTOPROTECTION IN MELASMA

Melasma is a refractory disorder and no single therapeutic approach has been proved to be entirely effective.^11^ Intensive and prolonged photoprotection is the cornerstone in melasma management.^12^, ^14^ Photoprotection includes minimizing sun exposure, particularly during midday; seeking shade; wearing wide‐brimmed hats, photoprotective clothing and sunglasses; applying sunscreen with sun protection factor (SPF) ≥30, and reapplying every 1–2 h when outdoors.^13^ Furthermore, patients must also be protected against HEVL and UVA1, which are more constant during the year and during the day.^13^

### 5.1 Use of sunscreens for prevention of melasma in high‐risk individuals

Pregnant women, especially those with higher skin phototypes, are at increased risk for the development of melasma. Incidence can be >50% in these individuals.^35^ A 12‐month clinical trial (CT) on 200 Moroccan parturients assessed the intensive use of a broad‐spectrum UVB–UVA sunscreen (SPF 50+, UVA‐PF 28) containing titanium dioxide. Only five new cases of melasma were among the 185 pregnant women who completed the study, corresponding to 2.7%, vs. an incidence of 53% observed in a previous study performed by the same investigators in the same time frame and region. Colorimetric data showed that the degree of epidermal pigmentation decreased or remained stable in 79% of the participants. No adverse events were reported.^35^ Similar results were observed in another 12‐month CT including 220 Korean parturients with skin phototype III and IV (*n* = 217). Patients were instructed on intensive use of a broad‐spectrum sunscreen (SPF 50+, UVA‐PF 30). Only 1% developed melasma, which was mild.^36^ In light of these findings, intensive photoprotection could be considered for individuals at high‐risk of developing melasma such as DSI during pregnancy, hormonal therapy or while taking oral contraceptives (Table 2).

**TABLE 2:** High‐risk factors for developing melasma

Sex/skin phototypes	Women of Fitzpatrick skin phototype III‐VI
Races/ethnic background	Asians and Latin Americans
Family history	Siblings and/or parents affected
Female sex hormone variations	Pregnancy Oral contraceptives use Hormonal therapy
Environmental factors	Chronic sun exposure

### 5.2 Sunscreens for melasma treatment

Regarding the use of sunscreens for treatment of melasma, a CT (n=100) assessed the use of a broad‐spectrum sunscreen (SPF 19 and PA+++) as the sole agent for the treatment of melasma in South Asians patients. There was both an objective and subjective improvement in melasma after 12 weeks of sunscreen use in terms of the Melasma Area Severity Score (MASI), and a significant improvement in quality of life.^10^ In a 12‐month CT, eight out of 12 parturients with preexisting melasma achieved marked clinical improvement with the intensive use of a broad‐spectrum UVB–UVA sunscreen (SPF 50+, UVA‐PF 28) in monotherapy.^35^

To assess the impact of protection against HEVL in melasma patients, a randomized CT (RCT) (n=68) compared two broad‐spectrum sunscreens SPF50. One of the sunscreens contained iron oxides and protected against HEVL (UVA/UVB‐HEVL). Both groups received 4% hydroquinone as a depigmenting therapy. The UVA/UVB‐HEVL group showed greater improvements in MASI scores, colorimetric values and melanin assessment. There was also a significant improvement in lightness values and a greater reduction in melanin content on histologic evaluation in patients applying UV/UVB‐HEVL sunscreen. The authors concluded that UVA/UVB‐HEVL sunscreens can enhance the depigmenting efficacy of topical hydroquinone.^37^ Another RCT compared the efficacy of three different sunscreens in preventing visible light‐induced pigmentation in 10 women with skin phototype IV. Two formulas were tinted and contained iron oxides at 4.85% or 27.25% (the former also contained titanium dioxide 27%) and the other was a non‐tinted mineral SPF 50+ sunscreen (containing titanium dioxide and zinc oxide). Expert grading and colorimetry showed that the iron‐oxide containing formulations significantly protected against VL‐induced pigmentation, with no differences between them, while the mineral SPF50+ sunscreen gave similar results to untreated skin.^38^ These findings highlight the importance of sunscreens containing iron‐oxide in protecting against VL, and their relevance for melasma treatment.

### 5.3 Sunscreens for preventing melasma relapses

Patients suffering from melasma have a high‐risk of clinical relapses.^11^ Prolonged use of photoprotection is highly recommended. An RCT (*n* = 40) compared two sunscreens in the prevention of melasma relapses. Both formulas contained the same filters against UV, although formula A was tinted (contained iron oxides) and protected against HEVL. The median increase of MASI score from baseline to month 6 was higher for the non‐tinted sunscreen than for the tinted sunscreen (*p *= .027). Eight patients in the non‐tinted sunscreen group used makeup during the trial. These individuals did not have fewer relapses than those using only non‐tinted sunscreen, highlighting the role of the specific protection against HEVL provided by iron oxides.^39^

### 5.4 Non‐filter active ingredients for sunscreen formulation

A wide range of active ingredients can be added to sunscreens to increase their efficacy in melasma management and/or tolerability to topical therapy. While a high proportion of ROS formation is secondary to UV radiation (4% attributed to UVB and 46% to UVA), it has been estimated that 50% of the generation of ROS can be attributed to VL exposure.^20^ The addition of antioxidants and free radical scavengers to sunscreens may help prevent some detrimental effects of VL, and may also protect against the damaging effects of infrared radiation, which is not specifically targeted by available sunscreens.^12^, ^20^ Several exogenous antioxidants and free radical scavengers have been identified, including vitamin E, vitamin C, diethylhexyl syringylidene malonate, Feverfew extract and the root extract of *Glycyrrhiza inflata*.^20^ The use of a topical antioxidant blend containing diethylhexyl syringylidene 2%, vitamin E 0.25% and vitamin C 0.01% have been shown to reduce pigmentation caused by VL+UVA1 in skin phototypes IV‐VI.^40^ Topical treatment with an SPF50 + sunscreen containing root extract of *Glycyrrhiza inflata* significantly reduced the VL‐induced depletion of intradermal carotenoids.^41^ Pretreatment with a broadband spectrum sunscreen containing Feverfew extract and other antioxidants significantly decreased oxidative stress in human subjects after VL irradiation.^18^ Although these findings are promising, further clinical studies are needed to establish the efficacy of this approach. Importantly, although oxidative stress induced by UVA and VL might play a role in the pathophysiology of melasma by altering the dermal component, it is well demonstrated that oxidative stress is not responsible for VL‐induced hyperpigmentation, which is secondary to the direct activation of the opsin 3 receptor at the surface of melanocytes by HEVL photons.^28^ Thus, adding antioxidants to sunscreen appears of interest, but cannot replace the use of a physical shield, such as iron oxides, which are the only ones that have demonstrated efficacy in reducing melasma severity and in preventing melasma relapses.^39^

While melasma skin presents with normal stratum corneum hydration and transepidermal water loss under basal conditions, it is characterized by impaired stratum corneum integrity and a delayed barrier recovery rate.^42^ It has been proposed that defects in the skin barrier function can lead to cutaneous pigmentation.^43^ As moisturizers can increase the moisture content of the epidermis and help restore the barrier function of the skin, the use of emollients/humectants and/or cosmeceuticals can be helpful for the management of melasma.^44^ Addition of these compounds to sunscreen formulas may increase tolerance and adherence to therapy.

Multiple depigmenting agents could improve the depigmenting properties of sunscreens in melasma. Niacinamide has antioxidant effects and suppresses skin pigmentation. An RCT (*n* = 27) compared niacinamide 4% versus hydroquinone 4% in the treatment of melasma. No difference in clinical response was found between both treatments.^45^ The combination of licorice extract and niacinamide was also effective in melasma treatment.^46^ Diverse resorcinol derivatives such as isobutylamido‐thiazolyl‐resorcinol, hexyl resorcinol, phenethyl resorcinol and 4‐n‐butyl resorcinol have been shown to be potent inhibitors of tyrosinase.^47^, ^48^, ^49^, ^50^ An RCT (*n* = 50), which compared isobutylamido‐thiazolyl‐resorcinol 0.2% vs. hydroquinone 4% for melasma treatment found no difference in clinical outcomes between both treatments, although contact dermatitis to isobutylamido‐thiazolyl‐resorcinol was reported in 8% of the participants.^51^ Sunscreens containing active depigmenting agents can be a novel alternative for the management of melasma, and can make therapy easier for patients.

### 5.5 Cosmetic elegance as a key factor for patient adherence

The cosmesis of sunscreen formulas can be a critical factor to ensure compliance. DSI can find the white residue from physical sunscreen unappealing and may reduce its application.^30^ A recent survey (*n* = 77) showed that dermatologists never, rarely, or only sometimes take patients’ skin phototype into account when prescribing a sunscreen, and valued cosmetic elegance as the least important factor when making recommendations for DSI.^30^ Ultra‐light texture, water‐based formulas can be excellent alternatives for encouraging the regular use of sunscreens by DSI (Table 3). As only tinted products containing a physical shield, such as iron oxides, which have shown up to 85% attenuation across wavelengths of 415 to 465 nm,^52^ have proven their efficacy in melasma, we must advise the use of these kinds of sunscreens. They not only provide protection against HEVL and UVA1, but can act as camouflage, and may facilitate social interactions and improve quality of life.^52^, ^53^ We must emphasize that the colors of currently available tinted sunscreens are far from being suitable for all skin tones; this applies to dark skin, including Asian skin, but also to very fair skin. The development of a wider variety of colors to match all skin tones is required.

**TABLE 3:** Characteristics of recommended sunscreens for melasma

Sun protection	UVB +UVA (including UVA1) + HEV (400–465 nm) ≥ SPF 30+ ≥ UVA‐PF 20
Formula texture	Easy to apply, non‐greasy, water‐based formulas. It should not leave white residue on the skin
Active ingredients (other than UV filters)	Should contain antioxidants, anti‐inflammatories and/or immunomodulators to enhance therapeutic action Depigmenting agents could improve clinical responses
Tinted sunscreens	Camouflage can impact positively on quality of life Tinted sunscreens containing formulations of pigmentary iron oxides can protect against HEV and UVA1 Various colors to match to most skin tones

Abbreviations: HEV, high‐energy visible light; UVA1, long‐wave UVA.

## 6 CONCLUSIONS

Treatment of melasma is challenging. Adequate and prolonged photoprotection is paramount. Intensive use of sunscreens can prevent melasma in high‐risk individuals, can improve melasma severity (associated or not with topical or systemic depigmenting agents), and can reduce relapses. Due to the pathophysiology of melasma, UVA/UVB protection alone is not sufficient. Sunscreens for this condition should be broad‐spectrum and high SPF, provide high protection against UVA1 and HEVL, and be cosmetically acceptable. Tinted sunscreens offering high SPF and high UVA‐PF and containing a physical shield such as iron oxides are the best option, and may provide camouflage. For patients with a skin tone, which does not match with the proposed colors of tinted sunscreens, one alternative is to use a non‐tinted sunscreen offering high UVB and UVA protection, and then apply a camouflaging makeup containing high concentration of iron oxides to offer HEVL protection. Recent advances in understanding the role of reactive species generated by VL may open the door to the incorporation of antioxidants to sunscreens to protect against the detrimental effects of VL on pigmentary disorders.

## CONFLICT OF INTEREST

DMC, JPC, and TP have received consultancy fees from ISDIN laboratories. CG, CT are ISDIN employees.

## Data Availability

The data that support the findings of this study are available on request from the corresponding author. The data are not publicly available due to privacy or ethical restrictions.
